# Alterations in urinary microbiota composition in urolithiasis patients: insights from 16S rRNA gene sequencing

**DOI:** 10.3389/fcimb.2023.1266446

**Published:** 2023-10-31

**Authors:** Haoran Liu, Qingqing Hu, Qunsheng Yan, Zongyao Hao, Chaozhao Liang

**Affiliations:** ^1^ Department of Urology, The First Affiliated Hospital of Anhui Medical University, Hefei, China; ^2^ Institute of Urology & Anhui Province Key Laboratory of Genitourinary Diseases, Anhui Medical University, Hefei, China

**Keywords:** urinary microbiota, urolithiasis, 16S rRNA gene sequencing, microbial diversity, clinical correlations

## Abstract

**Objectives:**

To investigate the urinary microbiota composition in urolithiasis patients compared to healthy controls and to identify potential microbial markers and their association with clinical parameters.

**Methods:**

A total of 66 samples, comprising 45 from urolithiasis patients and 21 from healthy controls, were analyzed. 16S rRNA gene sequencing was employed to determine the microbiota composition. Various statistical and bioinformatics tools, including ANOVA, PCoA, and LEfSe, were utilized to analyze the sequencing data and identify significant differences in microbial abundance.

**Results:**

No significant demographic differences were observed between the two groups. Post-quality control, clean tags ranged from 60,979 to 68,736. Significant differences in α-diversity were observed between the two groups. β-diversity analysis revealed distinct clustering of the urinary microbiota in urolithiasis patients and controls. Notably, Ruminococcaceae was predominant in urolithiasis samples, while Proteobacteria was more prevalent in healthy samples. Lactobacillus was significantly overrepresented in samples from healthy females.

**Conclusion:**

The urinary microbiota composition in urolithiasis patients is distinct from that of healthy controls. Specific microbial taxa, such as Ruminococcaceae and Proteobacteria, could serve as potential biomarkers for urolithiasis. The findings pave the way for further exploration of the role of microbiota in urolithiasis and the development of microbiome-based therapeutic strategies.

## Introduction

Urinary calculi is one of the commonest diseases in urology, impacts approximately 10% of the global population. Alarmingly, the recurrence rate within a decade is as high as 50%. Clearly, urolithiasis poses significant clinical challenges and carries a substantial economic and medical burden. Depending on the underlying cause, urolithiasis can be classified into two main types: metabolic calculi or infectious calculi.

The past few years have witnessed a multitude of breakthroughs in human microecology research, thanks to the swift advancement and implementation of high-throughput sequencing technologies. This progress has disrupted the traditional notion that organs not in direct contact with the outside world are sterile. The latest perspective suggests that the body’s microecology serves as a crucial organ, playing a significant role in preserving human health and preventing disease, a view gradually gaining acceptance in academic circles (O’Hara and Shanahan, 2006). Studies suggested that dysbiosis of body microbiome may cause disease progression (Darveau, 2010; Irrazabal et al., 2014; Chu et al., 2016; Urbaniak et al., 2016). Among the various systems within the human body, intestinal microecology has garnered significant attention. Conversely, research on urological microecology has seen a later start and consequently, less attention.

With the advent of novel technologies, new findings suggest the presence of resident flora within the urinary tracts of healthy individuals, playing an indispensable role in preserving urinary health. Traditional culture-dependent methods often used to detect uropathogens in urine samples have shown limited ability to identify a broader spectrum of microorganisms. This has led to an underestimation of the bacterial diversity within these communities. The urinary microbiome has been associated with various urological diseases, including genitourinary cancer, chronic prostatitis, and overactive bladder syndrome, among others (Shrestha et al., 2018; Li et al., 2019). However, the characteristics and functions of the urinary microbiome in patients with urinary calculi remain under-researched.

A recent population-based study indicated a significant correlation between exposure to multiple oral antibiotics and an increased incidence of nephrolithiasis (Tasian et al., 2018). This implies a potential role of microbiome dysbiosis in the pathophysiology of nephrolithiasis. Most research in this area, over the past few years, has focused primarily on the role of gut oxalate-metabolizing bacterial species in regulating urinary oxalate excretion. However, the most advanced perspective suggests that the urinary microbiome is more closely linked to urinary calculi than the gut microbiome (Zampini et al., 2019).

The principal aim of our current study was to characterize the urinary microbiota of patients with upper urinary tract stones. We aimed to investigate whether patient clinical characteristics are influenced by the composition of the urinary microbiome and explore the possible role of the microbiome in the pathogenesis of nephrolithiasis. We employed 16S rRNA gene sequencing technology to identify the urinary microbiome in stone formers. Subsequently, various bioinformatics analysis methods were utilized to predict their functional pathways.

## Methods

### Subject recruitment and urine specimen collection

From October to December 2022, we recruited 66 participants at The First Affiliated Hospital of Anhui Medical University, including 45 upper urinary tract calculi formers and 21 age-matched healthy volunteers who were undergoing routine physical examinations at the same institution. Urolithiasis patients were identified *via* ultrasonography, in combination with either computed tomography, abdominal plain film, or intravenous pyelography. The presence of urinary calculi was subsequently confirmed during endoscopic surgery, with the chemical composition of the calculi being analyzed through infrared spectroscopy.

We set stringent exclusion criteria to mitigate potential confounding factors that could distort the structure of the urinary microbiome. All healthy controls underwent ultrasonography to eliminate those with a personal history of urolithiasis. Additional exclusion criteria applied to all participants encompassed menstruation or pregnancy, age (below 18 or above 70 years old), diabetes, cancer, autoimmune diseases, benign urinary conditions, urinary tract malformations, chronic kidney disease with blood creatinine levels exceeding 1.4 mg/dL, urinary tract infections, or the use of antibiotics within the past four weeks. Urine samples from the participants were meticulously collected using the clean catch method. These samples were then immediately centrifuged at 12,000 g for 10 minutes at 4°C, and subsequently stored at -80°C pending further analysis.

Data collection procedures strictly adhered to the principles outlined in the Declaration of Helsinki. All participants provided written informed consent, permitting the use of their anonymous information in this study. The Medicine Institutional Review Board of Anhui Medical University granted approval for this study (LLSC20209856).

### Biochemical indicator detection and routine blood and urine analysis

Approximately 3 ml of whole blood samples were collected from each patient with urinary calculi for routine analysis and measurement of other biochemical metabolic parameters. The samples were centrifuged for 15 minutes at 3000 rpm at room temperature and stored at -80°C for further analysis. Baseline biochemical indicators of blood and urine samples were quantitatively determined by automatic biochemical analyzers, while other non-conventional indicators were tested using specialized assay kits as per standard procedures.

### DNA extraction and amplicon generation

Total genomic DNA was isolated using a DNA Extraction Kit in accordance with the manufacturer’s guidelines. The DNA concentration was validated using NanoDrop technology and agarose gel electrophoresis. The genomic DNA served as the template for PCR amplification with barcoded primers and Tks Gflex DNA Polymerase (Takara). The V3-V4 variable regions of 16S rRNA genes were amplified using the universal primers 343 F and 798 R.

### Library construction and sequencing

The quality of the amplicons was visualized using gel electrophoresis and purified with AMPure XP beads (Agencourt). This was followed by another round of PCR amplification. Post-purification with AMPure XP beads, the final amplicon was quantified using the Qubit dsDNA assay kit. Equal amounts of the purified amplicon were pooled for subsequent sequencing. Sequencing was carried out on an Illumina NovaSeq6000 platform, employing two paired-end read cycles of 250 bases each.

The sequencing raw data was uploaded in google drive and can be accessed via: https://drive.google.com/file/d/1gdoj0lIdLqD8Y-lXOngi_rjuHNpLXWPs/view?usp=sharing

### Bioinformatic analysis

Raw sequencing data were obtained in FASTQ format. Paired-end reads were preprocessed using Trimmomatic software to detect and remove ambiguous bases (N) and low-quality sequences with an average quality score below 20 through a sliding window trimming approach. Following trimming, paired-end reads were assembled using FLASH software, setting parameters as 10bp minimal overlap, 200bp maximum overlap, and a 20% maximum mismatch rate.

Further denoising of sequences was performed as follows: reads with ambiguous, homologous sequences or those below 200bp were discarded, while reads with 75% of bases exceeding Q20 were retained. Chimeric reads were then identified and eliminated, with these steps facilitated by QIIME software (version 1.8.0).

Clean reads were subjected to primer sequence removal and clustered to generate operational taxonomic units (OTUs) using Vsearch software with a 97% similarity cut-off. The representative read of each OTU was selected using the QIIME package. All representative reads were annotated and aligned against the Silva database Version 132 using the RDP classifier (confidence threshold was 70%). Unweighted Unifrac Principal coordinates analysis (PCoA) and phylogenetic tree construction were carried out using the Unifrac distance matrix generated by QIIME software.

### Statistical analysis

Statistical analyses were performed using SPSS (version 21.0) and R software (version 3.4.1), with P values < 0.05 deemed statistically significant. Clinical categorical variables were compared using Pearson’s chi-square test or Fisher’s Exact Test, while continuous variables were analyzed *via* a Student’s t-test. Age and body mass index were reported as mean ± standard deviation. For α-diversity and taxonomic analysis, the Wilcoxon rank-sum test or Kruskal-Wallis test were performed using R software. For β-diversity, comparisons of weighted UniFrac distances were conducted by ANOSIM using the vegan package of R software. ANOVA and Metastat method were applied to analyze the significantly different species among samples. PICRUSt software was used to predict the functional composition of known microbial genes, enabling the statistical comparison of functional differences between different samples and groups.

## Results

### General characteristics of urolithiasis patients and controls

We analyzed a total of 66 samples, with demographic and clinical data listed in **Table 1**. Urine specimens were collected from 45 urolithiasis patients and 21 healthy controls. As shown in **Table 1**, there were no significant differences (p>0.05) observed in demographic characteristics such as age, body mass index, gender, comorbidities, and history of smoking and drinking between the two groups. The prevalence of cardiovascular and cerebrovascular diseases, as well as diabetes, was relatively low among all subjects, while hypertension was a more common comorbidity.

**Table 1:** Demographic and clinical characteristics for subjects.

Characteristic	Total (n=66)		P value
	Stone patients (n=45)	Healthy control (n=21)	
**Age**	47.93 (14.32)	46.67 (15.32)	0.74
**BMI**	70.286(10.62)	69.167(1198)	0.72
**Gender**			0.81
Male	33	16	
Female	12	5	
**Smoking**			0.93
Yes	4	2	
None	41	19	
**Drinking Alcohol**			0.93
Yes	6	3	
None	39	18	
**Hypertension**			0.95
Yes	11	5	
None	34	16	
**Coronary heart disease**			0.95
Yes	2	―	
None	43	20	
**Cerebrovascular disease**			0.98
Yes	1	0	
None	44	21	
**Diabetes**			0.76
Yes	3	―	
None	42	20	

### 16S rRNA gene sequencing data

A total of 66 samples were included in the project. Post-quality control, the number of clean tags ranged from 60,979 to 68,736. After chimera removal, the range of valid tags was between 51,213 and 62,452. The average length of valid tags varied from 401.25 to 425.36 bp, and the number of OTUs in each sample ranged from 1,016 to 4,780.

**Figure 1:**
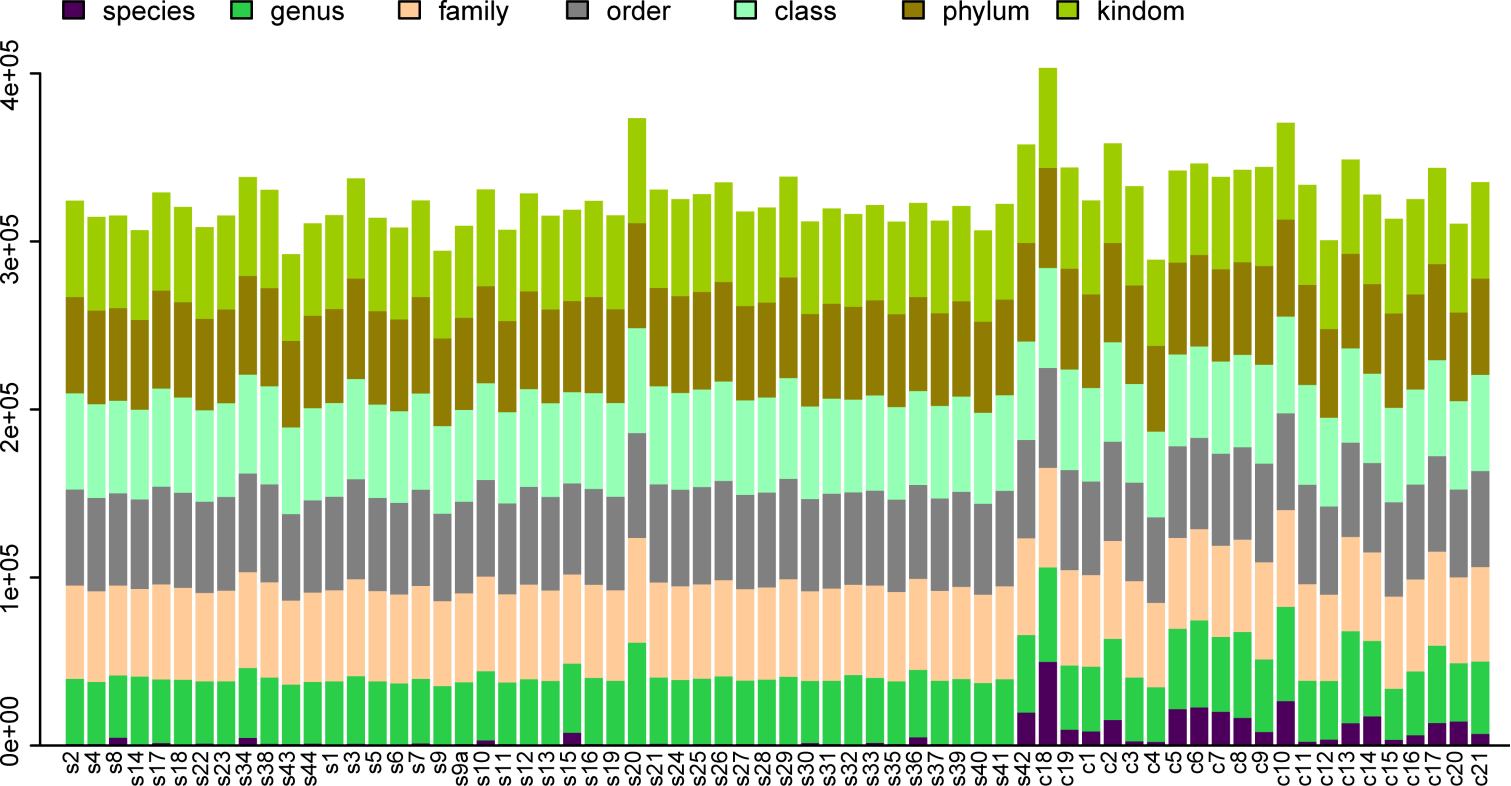
OTU Level Barplot. **(A)** The horizontal axis represents the sample name (with each column corresponding to a particular sample), while the vertical axis represents the total count of tags annotated at different classification levels on OTUs.

Differential statistics were computed for the project samples. According to the ANOVA algorithm, the numbers of differential OTUs, genera, and phyla were 4,122, 272, and 14, respectively. The total number of tags in OTUs at each level was summed to generate the OTU Level barplot representing the annotation ratio for each sample (**Figure 1**).

### Biodiversity of urinary microbiome

The alpha diversity of the microbiome was described using three indices: the Observed Species Index, the Chao1 Index, and the Shannon Index. The Observed Species Index represents the actual number of OTUs observed, while the Chao1 Index estimates the total count of OTUs within the sample. These two indices reflect the species richness of the urinary microbiota. The Shannon Index, on the other hand, indicates the species diversity of the urinary microbiome.

**Figure 2:**
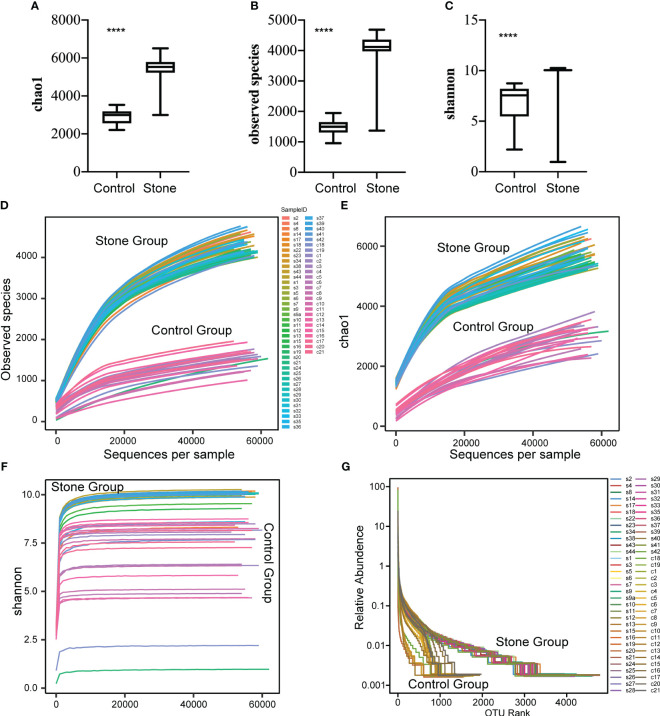
Alpha diversity of bacterial community in urine samples. The alpha diversity of the bacterial community in urine samples was assessed using the Observed Species Index **(A)**, Chao1 Index **(B)** and Shannon Index **(C)**. These indices were compared between samples from control subjects and patients with urinary stones. The mean values are represented by horizontal bars, while the error bars signify ± SD. The Wilcoxon rank-sum test was employed for the analysis. **(D–F)** Rarefaction curves were plotted for the observed OTU number and diversity indices. Each curve in the figure represents a sample. The depth of random sampling (i.e., the number of sequences sampled) is plotted along the x-axis, while the y-axis displays the exponential value. **(G)** The Rank Abundance Curve is depicted in Figure.c. OTUs are sorted along the x-axis from the most abundant to the least, based on the number of sequences each OTU contains. For instance, “500” signifies the OTU with the 500th highest abundance in the sample. The y-axis represents the relative abundance of the OTU, where “0.01” corresponds to 0.01%, and “0.1” represents 0.1%. (In these illustrations, “C” stands for control, “S” denotes stone, “C1” signifies female control, “C2” corresponds to male control, “S1” denotes female with stone, and “S2” represents male with stone.). **** p value < 0.001.

Significant differences (P<0.0001) were observed in the Observed Species Index, Chao1 Index, and Shannon Index (all representing alpha diversity) between the urine samples of patients with urinary calculi and healthy controls (**Figures 2A–C**). To determine if the sequencing volume of the samples was sufficient, we compared the rarefaction curves of different samples. These curves not only reflect whether the sequencing data volume is adequate to capture most microbial species information in the samples (**Figures 2D–F**), but also provide an intuitive contrast of species richness between samples. When the curve flattens or reaches a plateau, it indicates that the sequencing depth has nearly covered all microbiome species in the sample, and no further species can be detected by increasing the sequencing data.

We also plotted a Rank Abundance Curve to explain two aspects of microbial diversity: species abundance (i.e., the number of species in a community or habitat) and species evenness (i.e., the distribution uniformity of all species in a community or habitat) within the urine samples. The Rank Abundance Curve showed a greater diversity of microbial species in the urine from patients with urinary calculi compared to healthy individuals (**Figure 2G**). The species richness is reflected by the length of the curve along the horizontal axis: the wider the curve (the larger the span of the horizontal axis), the richer the species composition. Conversely, the shape of the curve reflects the uniformity of species composition: the flatter the curve (the smaller the vertical axis span), the more uniform the species composition.

**Figure 3:**
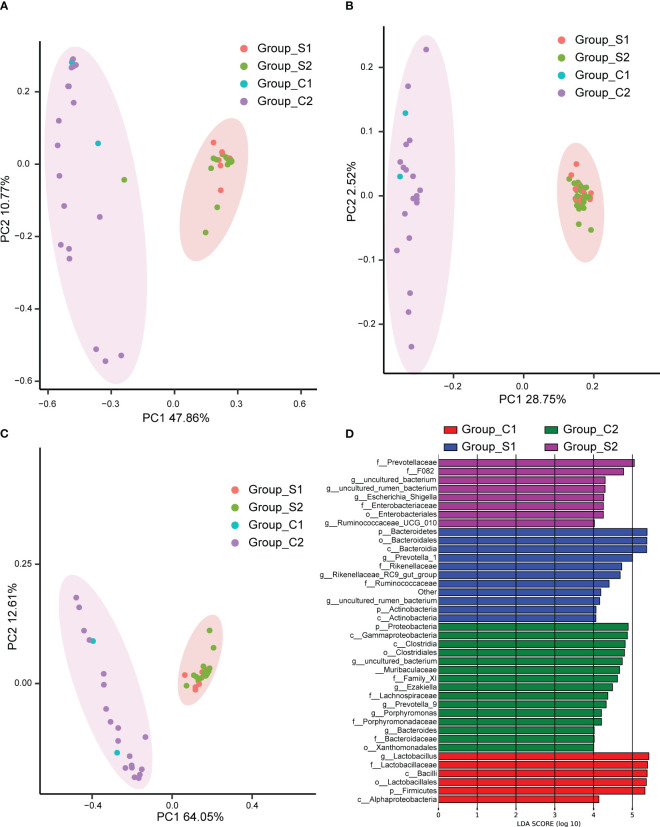
Principal Coordinate Analysis (PCoA) performed using Bray Curtis **(A)**, unweighted UniFrac **(B)**, and weighted UniFrac **(C)**. The abscissa (PC1) and ordinate (PC2) represent the two main coordinates, accounting for the largest proportion of the variance among samples. The same color indicates the same group, each point signifies a sample, and samples with similar characteristics cluster together. **(D)**The Linear Discriminant Analysis Effect Size (LEfSe) analysis of microbiomes among C1 (red), C2 (green), S1 (blue), and S2 (purple) groups. C1: female control; C2: male control; S1: female patients with stones; S2: male patients with stones.

In the investigation of group discrepancies, we employed Bray-Curtis, unweighted and weighted Principal Coordinate Analysis (PCoA) for the analysis of β-diversity. These methodologies consistently showed that the urinary microbiota in urolithiasis patients and the control group, irrespective of gender, clustered separately (**Figures 3A–C**). To assess the statistical significance of these distinct clusters, we conducted an ANOSIM analysis, which confirmed their significance (P < 0.05).

To further pinpoint specific taxa displaying significant differences in abundance between patients and controls, we utilized the LEfSe software. We set a logarithmic Linear Discriminant Analysis (LDA) score of 2 to discern significant differences. Consequently, we identified 40 taxa that differentiated among the groups, with several potentially serving as high-dimensional biomarkers for urolithiasis (**Figure 3D**). Most notably, the presence of Ruminococcaceae predominantly characterized urolithiasis samples, while Proteobacteria were more prevalent in healthy samples. Moreover, our results indicated a considerable overrepresentation of Lactobacillus in samples derived from healthy females.

### Urine microbiota composition

**Figure 4:**
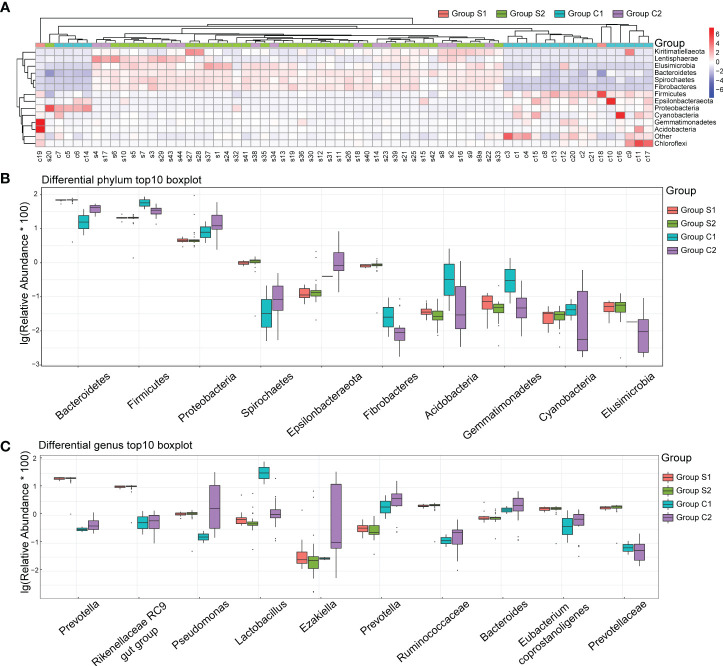
Urine Microbiota Composition. **(A)** Heatmap of Differing Species: The x-axis represents the sample numbers, while the y-axis provides the species annotation information. The species clustering tree is depicted on the left. The term “Group” in the cluster branch at the top refers to the samples originating from various groups. In the heatmap, red coloration indicates a higher relative abundance of species, whereas blue suggests a lower relative abundance. Each colored block in the heatmap corresponds to the abundance of a particular phylum within a sample. **(B)** Top 10 Boxplot of Species Abundance at Phylum Level: This plot visually represents the top ten species in terms of abundance at the phylum level. Different colors correspond to different sample groups. **(C)** Top 10 Boxplot of Species Abundance at Genera Level: This plot illustrates the top ten species concerning abundance at the genera level. Here too, distinct colors signify different sample groups. The y-axis shows the log-transformed values of species’ relative abundance. Please note, species with a zero relative abundance in a group are not displayed in these plots.

We constructed a heatmap based on the relative abundance of different species at the phylum level, allowing us to identify similarities in the composition of specific microbiota across samples (**Figure 4A**). The top 10 species with varied richness were selected for relative abundance boxplot analysis, enabling us to determine the intra-group abundance and inter-group comparisons of dominant species (**Figure 4B**). The results showed that Elusimicrobia, Bacteroidetes, Spirochaetes, and Fibrobactere were significantly more prevalent in the stone group than in the control group. Conversely, Firmicutes, Epsilonbacteraeota, Proteobacteria, Cyanobacteria, Gemmatimonadetes, and Acidobacteria were enriched in control individuals but decreased in urinary stone patients.

At the genera level, Prevotella, Rikenellaceae, Pseudomonas, Ruminococcaceae, Eubacterium Coprostanoligenes, and Prevotellaceae were significantly more abundant in the stone group (**Figure 4C**). In contrast, Lactobacillus, Ezakiella, Prevotella, and Bacteroides were significantly more abundant in the control group. Interestingly, gender did not significantly impact the distribution of bacteria in the urine.

### Correlations between clinical parameters and urine microbiome

**Figure 5:**
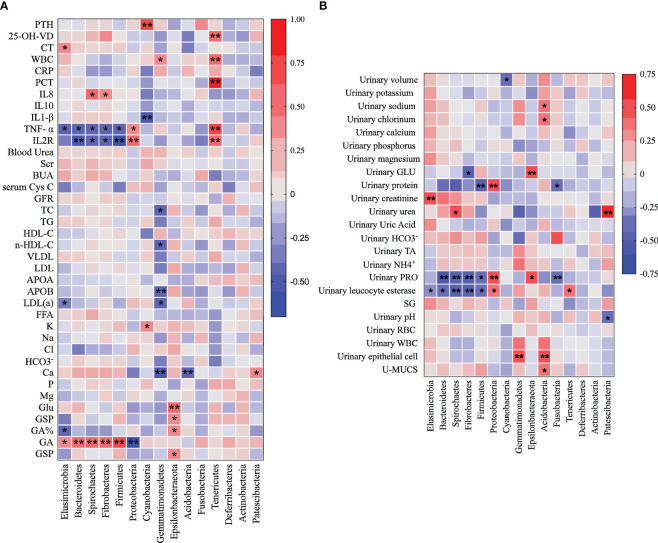
Heatmap illustrates the Pearson correlation analysis results between the relative abundance of distinct urine bacteria at the phylum level **(A)** and various clinical indicators **(B)**. Each cell in the heatmap represents a specific correlation value between a bacterial phylum and a clinical indicator. The significance of correlations is denoted by asterisks, where one asterisk (*) represents a p-value less than 0.05 and two asterisks (**) indicate a p-value less than 0.01.

Investigating the potential associations between substances found in blood and urine and the urine microbiome, we conducted correlation analyses at the phylum level for all urolithiasis patients. This revealed a myriad of significant links between metabolic and inflammatory indicators and the top ten enriched microorganisms in patients’ urine. PTH, CT, and 25-OH-VD, all related to calcium metabolism, were positively correlated with Cyanobacteria, Elusimicrobia, and Tenericutes, respectively (**Figure 5A**). Conversely, the calcium ion demonstrated a negative relationship with Gemmatimonadetes and Acidobacteria, while showing a positive association with Patescibacteria.

In other notable findings, Acidobacteria exhibited a positive correlation with 24-h urinary sodium and chlorinum, while Patescibacteria was positively related to 24-h urinary urea and negatively to urinary pH. Bacteroidetes, Spirochaetes, Fibrobacteres, and Firmicutes showed negative correlations with TNF-α, IL2R, urinary protein, and urinary leucocyte esterase. Glycated Albumin (GA) was positively related to Elusimicrobia, Bacteroidetes, Spirochaetes, Fibrobacteres, and Firmicutes.

Interestingly, Proteobacteria demonstrated a positive relationship with TNF-α, IL2R, urinary protein, and urinary leucocyte esterase, but was negatively correlated with GA. Gemmatimonadetes showed a negative correlation with TC, n-HDL-C, APOB, and LDL(a), whereas Epsilonbacteraeota exhibited a positive correlation with Glu, GSP, GA%, 24-h urinary GLU, and urinary protein.

Tenericutes, Cyanobacteria, and Elusimicrobia each displayed their unique correlation profiles. Tenericutes showed positive correlations with WBC, PCT, 25-OH-VD, TNF-α, IL2R, and urinary leucocyte esterase. Cyanobacteria had a negative relationship with IL1-β but was positively associated with PTH and K. Elusimicrobia exhibited a positive correlation with CT, GA, and 24-h urinary creatinine, but a negative correlation with TNF-α and urinary leucocyte esterase (**Figure 5B**). These correlations indicate a potential interplay between urine microbiota and various clinical parameters.

### Exploring metabolic biosynthesis pathways linked to the urinary microbiome

In our quest to understand whether the functional dynamics of the urinary microbiome in urolithiasis patients has shifted, we utilized the PICRUSt tool. This tool allowed us to predict the functional components present in the 16S rRNA gene sequencing data of all samples. Intriguingly, we observed a significant discrepancy in the functional structure between the stone patient group and the control group.

**Figure 6:**
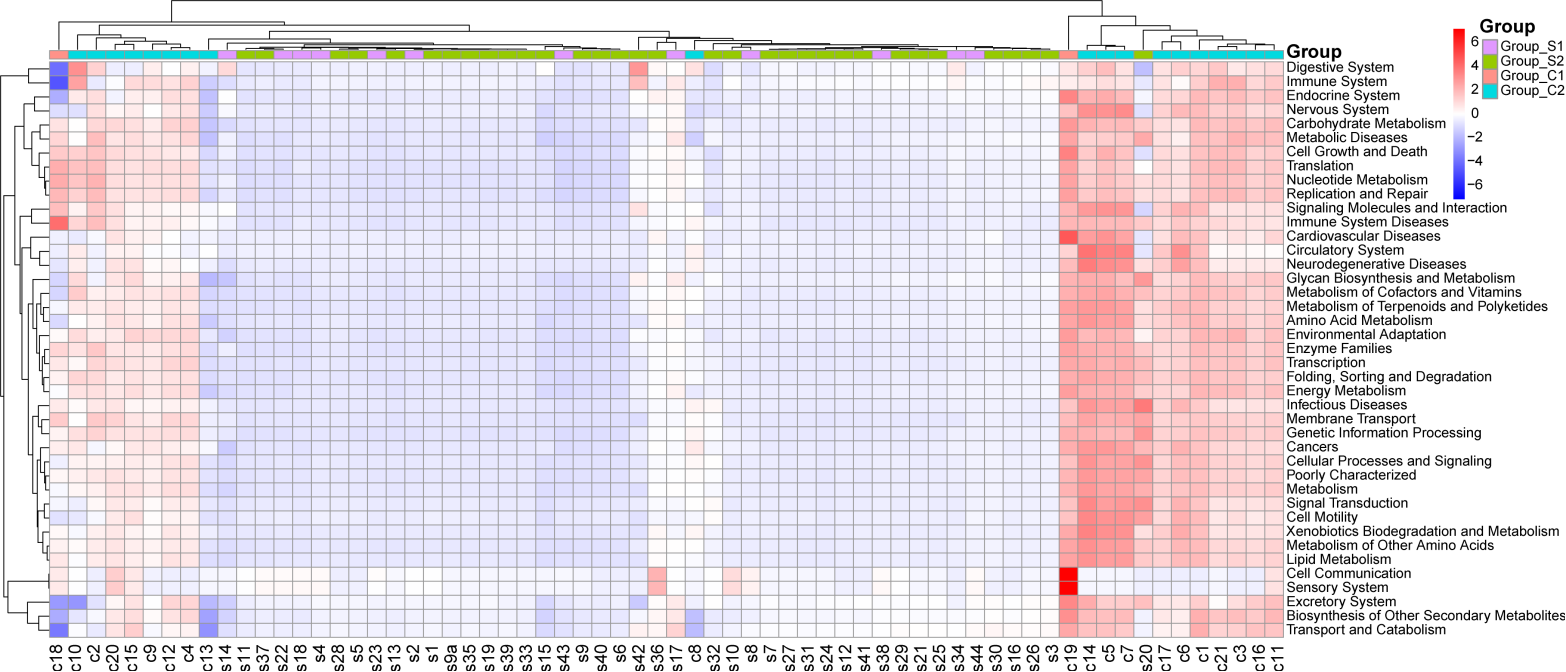
This figure presents a comparison of level 2 KEGG pathways between urolithiasis patients and healthy controls.

In contrast to healthy controls, nearly all predicted level 2 KEGG metabolic pathways were noticeably underrepresented in the stone group. However, specific pathways from the level 3 KEGG database, including protein digestion and absorption, isoflavonoid biosynthesis, glycosphingolipid biosynthesis - ganglio series, and glycosaminoglycan biosynthesis-chondroitin sulfate, were significantly over represented in the stone patient group (**Figure 6**).

Moreover, we discovered that gender did not contribute to the variation in our findings. This indicates that there was no significant difference in the predicted results between male and female subjects, regardless of whether the samples originated from healthy controls or urolithiasis patients.

## Discussion

The critical role of the human microbiota in mediating health and disease states is unequivocal. Groundbreaking developments in sequencing methodologies have facilitated thorough exploration of the remarkably complex microbial ecosystems colonizing various body sites, including the urinary tract (Casper et al., 2018). Traditionally, urine in healthy individuals was believed to be sterile, yet numerous enlightening studies using extensive quantitative urine culture and 16S rRNA gene sequencing have definitively revealed the presence of diverse and multifaceted microbial communities in urine, even without clinically detectable infections (Nissen, 2018). The specific composition and functional activities of the urinary microbiome seem to diverge dramatically between healthy individuals and patients with different urinary conditions, indicating possible pathogenic or protective functions of certain urinary microbial profiles, or urotypes (Vannella et al., 2014).

In our investigation, we found that urine samples from patients with kidney stones exhibited significantly higher microbial richness and diversity than samples from healthy controls, as indicated by greater observed species counts, Chao1 index, and Shannon index (Song et al., 2017). These key findings not only corroborate but also substantially extend previous studies that revealed increased urinary microbiome diversity in other disorders like interstitial cystitis, urinary incontinence, and prostate inflammation (Gaudesi et al., 2014; Nissen, 2018). At the phylum level, we noticed marked overrepresentation of Elusimicrobia, Bacteroidetes, Spirochaetes, and Fibrobacteres in patients with kidney stones, a finding that resonates with earlier groundbreaking studies associating gut microbiota alterations with a heightened risk of kidney stone formation (Bayefsky et al., 2016). In stark contrast, phyla such as Firmicutes, Epsilonbacteraeota, Proteobacteria, Cyanobacteria, Gemmatimonadetes, and Acidobacteria were significantly more abundant in healthy individuals (Keck et al., 2018).

Intriguingly, our study demonstrated a significant increase in Lactobacillus in healthy females, solidifying the proposed protective impact against urinary stone formation (Gaudesi et al., 2014; Alabi et al., 2017). The abundance of Lactobacillus has been strongly correlated with a decreased incidence of urinary tract infections (UTIs), emphasizing its critical role in preserving urinary health (Aflatoon et al., 2003; Sanchez-Andrea et al., 2012). Lactobacillus acts as a powerful inhibitor of uropathogens and beneficially modulates immunity through multiple mechanisms including lactic acid production, biosurfactants, hydrogen peroxide, bacteriocins, and adhesive exclusion. Increasing recognition is being given to the intricate regulation of the urinary tract’s biochemical environment by the complex interactions among diverse bacterial metabolites (Romero-Saavedra et al., 2014).

At the genus level, our data showed that Prevotella, Rikenellaceae, Pseudomonas, and Eubacterium coprostanoligenes were markedly more common in individuals with kidney stones (Vannella et al., 2014). Pseudomonas has a well-established association with UTIs and has frequently been isolated from urinary stones, suggesting possible pathogenic roles (Romero-Saavedra et al., 2014). Noteworthy studies have reported a predominance of Pseudomonas in certain bladder cancer patients, highlighting the urgent need to comprehensively identify urotypes associated with specific diseases (Vannella et al., 2014).

Our compelling Principal Coordinates Analysis (PCoA) results revealed distinct clusters of urinary microbiota composition between individuals with kidney stones and healthy controls, suggesting possible shared microbial signatures profoundly impacting the pathogenesis of urolithiasis. Nonetheless, growing awareness suggests that urinary microbiota’s extensive inter-individual variation could be influenced by factors such as genetics, diet, medication, and a variety of environmental factors (Gaudesi et al., 2014). Identification of robust molecular and functional hallmarks of “healthy” and “dysbiotic” urotypes could provide transformative insights into stone pathogenesis and guide personalized therapeutic strategies (Nissen, 2018).

Unraveling the causal relationship between the urinary microbiome and kidney stone formation will require integrating multi-omics approaches with animal models and prospective clinical studies. Such efforts will foster the development of next-generation diagnostic and therapeutic applications (Kennedy et al., 2012). A comprehensive definition of healthy versus dysbiotic urotype signatures could play a crucial role in spurring microbiome-based interventions for recurrent nephrolithiasis.

In summary, our research underscores significant alterations in the urinary microbiome of individuals with kidney stones compared to controls. Further studies are critically needed to clarify causal links and explore therapeutic targeting of urotypes for nephrolithiasis prevention and treatment. Integrating varied omics data with clinical metadata will be instrumental in deciphering functional microbiome changes underpinning urolithiasis. The precise characterization of healthy and dysbiotic urotypes could potentially pave the way for personalized microbiome-based therapies for recurrent kidney stone formation.

## Data availability statement

Data supporting the results and raw data of this study can be obtained from NCBI: https://www.ncbi.nlm.nih.gov/bioproject/PRJNA1032262.

## Ethics statement

The studies involving humans were approved by The Medicine Institutional Review Ethics Board of Anhui Medical University. The studies were conducted in accordance with the local legislation and institutional requirements. The participants provided their written informed consent to participate in this study.

## Author contributions

HL and QH were responsible for conceptualization, writing, data analysis, and figure. QY was responsible the samples collection and data analysis. ZH and CL provided funding and conceptual support. All authors read and approved the final manuscript.
